# Toxicity of AMPA to the earthworm *Eisenia andrei* Bouché, 1972 in tropical artificial soil

**DOI:** 10.1038/srep19731

**Published:** 2016-01-21

**Authors:** Anahí Domínguez, George Gardner Brown, Klaus Dieter Sautter, Cintia Mara Ribas de Oliveira, Eliane Carvalho de Vasconcelos, Cintia Carla Niva, Marie Luise Carolina Bartz, José Camilo Bedano

**Affiliations:** 1 CONICET – Geology Department, National University of Rio Cuarto, 5804 Rio Cuarto, Argentina; 2 Embrapa Florestas, 83411-000 Colombo-PR, Brazil; 3 Universidade Positivo, 81280-330 Curitiba-PR, Brazil; 4 Embrapa Cerrados, 73310-970, Brasília-DF, Brazil

## Abstract

Aminomethylphosphonic acid (AMPA) - one of glyphosate’s main metabolites - has been classified as persistent in soils, raising concern regarding the widespread use of glyphosate in agriculture and forestry. Glyphosate may have negative or neutral effects on soil biota, but no information is available on the toxicity of AMPA to soil invertebrates. Therefore our aim was to study the effect of AMPA on mortality and reproduction of the earthworm species *Eisenia andrei* using standard soil ecotoxicological methods (ISO). Field-relevant concentrations of AMPA had no significant effects on mortality in acute or chronic assays. Except at the highest concentration tested, a significant biomass loss was observed compared to controls in the chronic assay. The number of juveniles and cocoons increased with higher concentrations of AMPA applied, but their mean weights decreased. This mass loss indicates higher sensitivity of juveniles than adults to AMPA. Our results suggest that earthworms coming from parents grown in contaminated soils may have reduced growth, limiting their beneficial roles in key soil ecosystem functions. Nevertheless, further research is needed to better understand the mechanisms underlying the sublethal effects observed here.

Glyphosate was first introduced in crop production in 1971^1^. However, its use expanded worldwide during the 1990’s, when herbicide-resistant, genetically-engineered crops allowed widespread use by farmers of broad-spectrum herbicides such as glyphosate^2^. Genetically modified (GM) crops now cover 175 million hectares in 27 countries worldwide, but 77% of that area is located in only three countries: USA (40%), Brazil (23%) and Argentina (14%)^3^. The increased use of GM crops has been accompanied by a concomitant increase in glyphosate use. In Argentina, 238 million litres of glyphosate were sprayed in 2011, and in Brazil, approximately 340 million litres, raising concerns of the possible non-target effects of this herbicide, especially its potential impact on soil and water contamination and ecosystem functioning^4^,^5^.

Earthworms are one of the most important components of the soil biota in terms of soil formation and maintenance of soil structure and fertility^6^. Furthermore, they are frequently used as indicators of soil quality and contamination levels, with standard, internationally recognized and adopted ecotoxicology assays^7^,^8^,^9^. These methods include acute and chronic tests. The former evaluates short-term and lethal effects of a potentially toxic agent, providing information on possible quick and dramatic changes in earthworm communities. On the other hand, chronic tests evaluate sub-lethal responses in longer-term parameters that are often more sensitive to soil pollution, such as growth and reproduction.

Although glyphosate effects on earthworms have been extensively studied, results are far from conclusive. Several studies consistently found very low mortality of *Eisenia andrei* Bouché, 1972 and *Aporrectodea* spp. worms at recommended (from 1,440 g ai.ha^−1^ to 1,800 g ai.ha^−1^) and higher doses of glyphosate^10^,^11^,^12^,^13^,^14^,^15^,^16^. García-Torres *et al.*^17^ reported significant mortality only at very high concentrations (50,000 mg.kg^−1^), but Piola *et al.*^18^ found that lethal doses depended on the commercial formulations used.

Conversely, sub-lethal parameters such as reproduction have usually been more sensitive than mortality in assessing glyphosate effects. For example, the number of juveniles and/or cocoons decreased at doses of 1 to 1,000 mg.kg^−1^, 5,000 mg.kg^−1^ and 1,440 mg ai.ha^−1^ 11,12,17. However, other studies found no significant effects of glyphosate on earthworm reproduction^10^,^13^,^15^,^19^. Hence, the risk of non-target toxic effects of glyphosate in soils appears to be low, particularly considering its’ short half-life: only 5 to 23 days^20^,^21^,^22^ in field conditions, although the degradation pattern is significantly affected by factors such as soil texture, pH, rain events and microbial activity^23^.

The major breakdown products of glyphosate are aminomethylphosphonic acid (AMPA) and sarcosine^20^,^24^. However, unlike glyphosate, AMPA has been classified as persistent in soils, with a typical half-life of 151 days, but varying from 76 to 240 days depending on field conditions^22^. The longer persistence of AMPA might result in higher toxicity risks compared to glyphosate, although very little information is available concerning AMPA toxicity^25^,^26^,^27^. To our knowledge, the effect of AMPA on soil invertebrates has not been previously studied. Therefore, the aim of this paper was to study the effect of AMPA on mortality and reproduction of the standard ecotoxicological test earthworm species *E. andrei*^8^ in tropical artificial soil (TAS).

## Results

### Acute toxicity test

**Figure 1:**
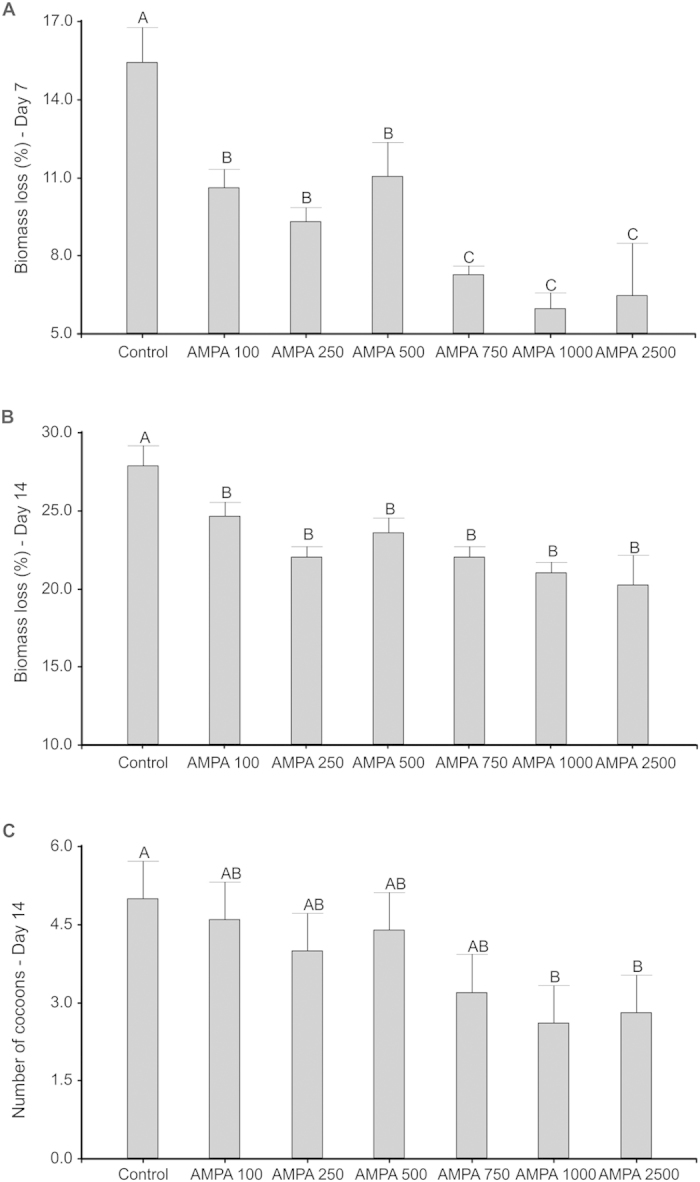
Effects of exposure to different doses of AMPA (100, 250, 500, 750, 1,000 or 2,500 μg.kg^−1^) in tropical artificial soil on *Eisenia andrei* growth (presented as biomass loss, in % of initial weight) after 7 days (A) and 14 days (B) and on the number of cocoons (C), using the ISO (2012) standard acute toxicity test^8^. Different letters denote significant differences between treatments (p < 0.05).

No significant mortality was observed in the control and in any of the tested AMPA concentrations. Only two earthworms died in the 500 μg.kg^−1^ treatment. Biomass loss (Fig. 1A) at day 7 was lowest (5.98–7.26%) at the three highest doses (750 to 2500 μg.kg^−1^), intermediate (9.32–11.05%) at the lowest doses (100 to 500 μg.kg^−1^), and highest (15.41%) in the control. At day 14 (Fig. 1B) biomass loss was higher in the control (29.7%) than in all other treatments, and similar regardless of the AMPA dose applied. The higher mass loss in the control treatment was accompanied by energy and biomass investment in reproduction, as cocoon production was highest in the control and significantly higher when compared to the AMPA1000 and AMPA2500 treatments (Fig. 1C).

### Chronic toxicity test

**Figure 2:**
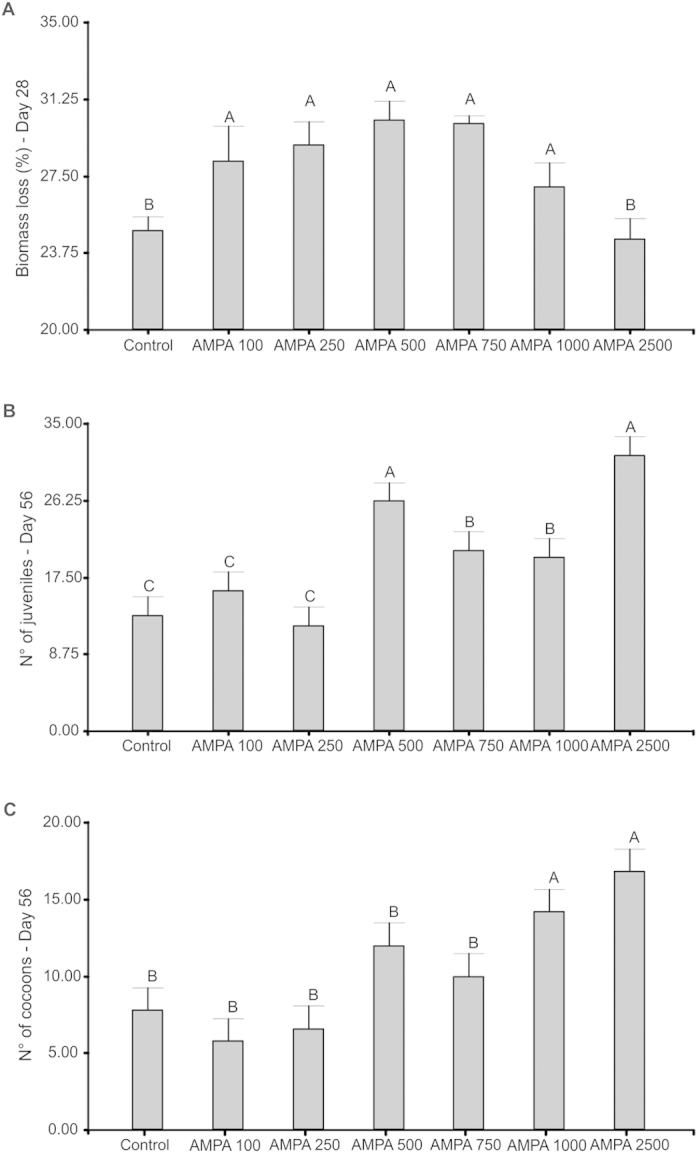
Effects of exposure to different doses of AMPA (100, 250, 500, 750, 1,000 or 2,500 μg.kg^−1^) in tropical artificial soil on *Eisenia andrei* growth (presented as biomass loss, in % of initial weight) after 28 days (A), and on the number of juveniles (B) and cocoons (C) after 56 days, using the ISO (2012) standard chronic toxicity test^8^. Different letters denote significant differences between treatments (p < 0.05).

**Figure 3:**
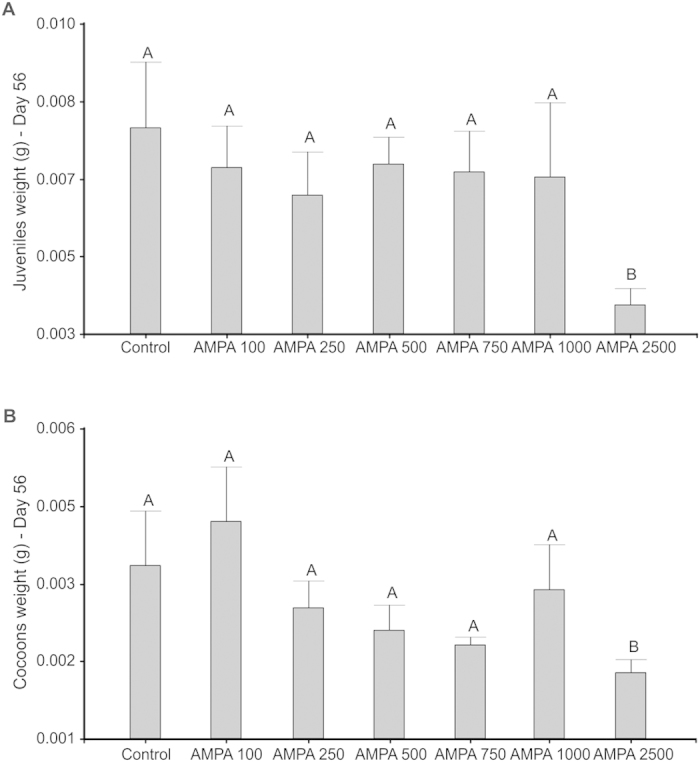
Effects of exposure to different doses of AMPA (100, 250, 500, 750, 1,000 or 2,500 μg.kg^−1^) in tropical artificial soil on the fresh weight of *Eisenia andrei* juveniles (A) and cocoons (B). Different letters denote significant differences between treatments (p < 0.05).

No significant mortality was found in control and in all other treatments, and only one earthworm died in the AMPA2500 treatment after 28 days. In contrast to the acute assay (above), biomass loss (Fig. 2A) was significantly lower in the control treatment compared to all the AMPA treatments, except for AMPA2500. In the control soil, 13.2 juveniles were found and 7.8 cocoons on average (Fig. 2B,C) after 56 days, with no significant difference from the values found in the AMPA100 and AMPA250 treatments. In all the higher AMPA doses, the number of juveniles was significantly higher than in the control, and at the highest AMPA doses (1,000 and 2,500 μg kg^−1^) the number of cocoons was also higher. However, the mean weights of both juveniles and cocoons were significantly lower in AMPA2500 than in all other treatments (Fig. 3A,B).

## Discussion

According to ISO guideline 11268-1^8^ the results of the mortality assays are valid when the mortality of adult worms in the controls is ≤10%. This criterion was met in the present study (0% mortality). According to ISO guideline 11268-2^8^ the results of the reproduction assays are valid if the percentage of mortality of the adults observed in the controls is ≤10%, if the coefficient of variance (CV) of reproduction in the control does not exceed 30%, and if the rate of production of juveniles is at least 30 per control container. The first two criteria were fulfilled but the minimum rate of 30 juveniles per control container was not met (n = 13 juveniles in the present study). Nevertheless, as the latter criterion is often not met in control soils/substrates^11^,^12^, we considered the present results as valid and report them here.

The toxicity of the reference substance used (boric acid) was according to the expected. No cocoons or juveniles were produced in both the acute and chronic assays. In the long term assay, biomass loss was significantly higher compared with the control, as has been observed in other long term assessments^28^. Thus, the effect on reproduction was similar to that found by Becker *et al.*^29^ at concentrations of 750 and 1,000 mg.kg^−1^ boric acid. Therefore, both the sensitivity of the *E. andrei* specimens used and the laboratory test conditions were considered adequate, validating the present tests.

The absence of significant mortality in all the AMPA doses tested is consistent with most studies on glyphosate acute effects on *E. fetida* and *E. andrei*^10^,^11^,^12^,^13^,^17^. Therefore, field application rates and field concentrations of AMPA up to 2,500 μg.kg^−1^ should not cause significant mortality of *E. andrei* in the short term (up to 28 days). Nevertheless, this does not discard the possibility of significant effects of AMPA on the survival of other earthworm species, since not all earthworm species respond in the same manner to soil contamination^5^. Nevertheless, it appears unlikely that there will be major effects on acute toxicity, considering the results already available for glyphosate and different earthworm species^10^,^11^,^12^,^13^,^17^.

In fact, this is the first assessment of AMPA toxicity on a soil organism. AMPA acute toxicity has been described to be low for rats and moderate for fish and aquatic invertebrates^22^. In the fish *Anguilla anguilla* genotoxic damage was also shown^27^, while in amphibians, the residence time in water was not significantly affected with doses up to 500 μg.L^−1^ 30.

However, acute assays are generally less sensitive than chronic assays^31^, which provide more realistic results on long-term sublethal effects, based on growth and reproduction. For instance, the acute assay did not reveal any negative effects of AMPA on biomass, and in fact earthworms in AMPA treatments after 7 and 14 days had lower biomass losses than earthworms in the control. On the other hand, at 28 days in the chronic test, biomass losses were significantly higher in all AMPA treatments (except the highest dose - AMPA2500) compared to the control.

The higher biomass loss in the acute test controls may be explained by the higher biomass investment in cocoon production in this treatment, particularly compared to the highest AMPA doses (1,000 and 2,500 μg.kg^−1^). Another possible explanation could be that AMPA can be used as an extra source of food for microbiota^32^ and could benefit earthworm growth. Indeed, high application rates of glyphosate have been found to stimulate microbial respiration^33^,^34^,^35^, since glyphosate is a P-containing aminoacid that functions both as a sole P source for *in vitro* microbial growth and as a readily available C and N source when degraded in soil. Therefore, the same could be expected for AMPA. However, only very high glyphosate doses (5,000 mg.kg^−1^) have been found as capable to significantly enhance microbial growth^32^. Moreover, our results tend to agree more with other studies that did not observe any significant effects of glyphosate on biomass loss, at concentrations of up to 200 mg.kg^−1^ 10,14,19.

No significant mortality in the long term reproduction assay was found, which also agreed with the fore-mentioned studies on glyphosate toxicity to earthworms and on AMPA toxicity to other organisms. On the other hand, biomass losses in earthworms exposed for 28 days to AMPA doses from 100 to 1,000 μg.kg^−1^ were significantly higher than in the control treatment. These results agree with Correia & Moreira^12^ and Yasmin & Souza^16^, who found negative effects of glyphosate at concentrations from 8 to 1000 mg.kg^−1^ on earthworm biomass, especially in the long term. However, there seems to be a threshold effect on earthworm growth, possibly somewhere in between the doses of 750 and 1,000 μg.kg^−1^ AMPA, since at 1,000 μg.kg^−1^ biomass loss was lower, and at the highest dose (2,500 μg.kg^−1^) biomass loss was similar to the control and lower than all other AMPA doses. As observed in the acute trial, the biomass loss in the low to intermediate AMPA doses appear to be related to a higher investment in reproduction by the earthworms in these treatments. Long term exposure to AMPA to higher but non-lethal concentrations, seems to produce higher stress which is reflected both by an increase in cocoon production and by a higher biomass loss compared to the control.

Oxidative stress has been proposed as one possible mode of action of glyphosate in non-target organisms^36^. Activity changes of biotransformation system enzymes have also been used as indicators of sub-lethal impact of pollutants. In this sense, Contardo-Jara *et al.*^36^ found an increase in soluble glutathione S-transferase (GST) in the blackworm *Lumbriculus variegatus*, at glyphosate doses ranging from 50 to 1,000 μg.L^−1^. Interestingly, they also found a threshold between 1,000 μg.L^−1^ and the highest dose tested: 5,000 μg.L^−1^. Furthermore, they found no difference in soluble GST activity at 5,000 μg.L^−1^ and the control treatment. Therefore, it is appears that the mechanisms by which AMPA exerts its toxicity on earthworms is different at high doses compared to intermediate and lower doses. Furthermore, the relationship between biomass loss and cocoon production appears to be different at the highest doses tested compared to the intermediate ones, suggesting that modes of action of AMPA at high doses are different for these two life-cycle parameters.

The number of juveniles found in the control soil after 56 days was greater than in Casabé *et al.*^11^, Correia & Moreira^12^ and García-Torres *et al.*^17^, but lower than in Santos *et al.*^15^. Thus, the values we found were among those expected for *E. andrei* but with some variations compared to previous studies. The AMPA100 and AMPA250 treatments did not increase the number of juveniles compared to control, but at all higher doses, a significantly higher number of juveniles and a progressively higher number of cocoons were observed. A hormesis effect of glyphosate could be involved in these results. Hormesis is the phenomenon in which sub-harmful levels of a stress agent may help an organism in suboptimal environments^37^. In some cases, pesticides have been shown to increase total fecundity, fecundity dependent on dose, or advance fecundity to an earlier age^37^. Still, few studies have found a stimulation of cocoon production at low or intermediate doses of contaminants such as Pb, Al and Cu, which are toxic at higher doses^38^. For instance, in their review of pesticides effects on earthworms Yasmin & Souza^31^ did not find any study reporting greater cocoon or juvenile production with increasing doses compared with controls. And while Gaupp-Berghausen *et al.*^39^ found a substantial decrease in cocoon production of two earthworm species (*Lumbricus terrestris* and *Aporrectodea caliginosa*) due to herbicides with glyphosate as active ingredient, Santadino *et al.*^40^ observed a significant increase in the number of *E. fetida* cocoons with increasing glyphosate doses. This result agrees with those of the present study and may be related to a hormesis effect. They observed lower fertility of those cocoons while we also found that the higher juvenile and cocoon production in the highest dose (AMPA2500) occurred together with a decrease in juvenile and cocoon biomass. Further studies including chemical and physiological evaluations will help reveal the mechanisms by which the higher stress produced by AMPA is transferred to a high juvenile and cocoon production, and how this is related to biomass loss.

The lower biomass found in cocoons and juveniles with higher AMPA doses might also be related to a higher sensitivity of juveniles than adults to AMPA, which would explain lower growth of juveniles, as has been previously observed^31^. Moreover, the production of more cocoons but lighter -and therefore presumably weaker- individuals, would be associated to weaker juveniles. This suggests that earthworms growing in soils contaminated with high doses of AMPA could have a lower physiological ability to develop, grow and reproduce in these soils and to accomplish key ecosystem functions.

## Conclusions

In the present study, the toxicity of glyphosate’s main metabolite – AMPA – to the earthworm *E. andrei* was studied in field-relevant concentrations. In both acute and chronic assays no significant mortality was recorded. Biomass loss in the short term assay was higher in the control compared to AMPA-contaminated treatments, probably due to energy and mass investment in higher cocoon production. However, in the long term assay, biomass loss was higher in AMPA treatments than in the control, except for the highest concentration in which a high production of significantly lighter cocoons and juveniles was recorded. This could be considered a reproduction disorder caused by AMPA. Hormesis is proposed as a possible mode of response of earthworms to AMPA, but further studies are needed to better understand the mechanisms and the physiology of AMPA toxicity to earthworms, and the role of low to intermediate contamination levels of AMPA in soils on earthworm growth and reproduction. This is especially important when considering the higher persistence of AMPA in soils compared to glyphosate, and the possible effects of long term exposure to high, but sub-lethal AMPA concentrations on earthworm populations and their potential roles in soil functioning.

## Materials and Methods

### Test species

*E. andrei*, the recommended species for ecotoxicological assessment with earthworms in guidelines for testing on chemicals^8^,^9^, were purchased from Minhobox (Juiz de Fora, Minas Gerais, Brazil), an earthworm breeder who guarantees specimens free of any previous contamination. Before each assay, earthworms were maintained for 24 h in a box with uncontaminated substrate, to allow acclimatization to the laboratory conditions and to the artificial substrate. Only adult (with clitellum) specimens with 250 to 600 mg live weight and normal morphology, and responding to mechanical stimuli were used.

### Test substrate

The assays were performed in tropical artificial soil (TAS), prepared according to García^41^. The TAS was a mixture of 70% fine sand, 20% kaolinite clay and 10% powdered coconut fibre, in replacement of sphagnum peat used in temperate artificial soils. Before mixing all the components, the sand was washed, air dried and sieved at 2 mm. Coconut fibre was also sieved (2 mm) and dried (60 °C). All components were mixed until 40 kg of homogeneous substrate was obtained for all the assays (2 assays × 8 treatments × 5 replicates × 0.5 kg). Final pH was always within the optimal range determined by ISO guidelines, i.e., 6.0 ± 0.5, so adjustment with calcium carbonate was not necessary. Water holding capacity of the TAS was measured and moisture content maintained at 60% of water holding capacity during the entire period of each assay by monitoring weekly changes in individual vessel weights. The day before beginning each assay, the TAS was pre-moistened with deionized water corresponding to 30% of the water holding capacity. The remaining water was used to dilute the AMPA.

### Test substance

AMPA (99% pure) was purchased from Sigma-Aldrich Co. (USA). The concentrations used in the assays were defined by field-relevant concentrations. To the best of our knowledge, 2,256 μg.kg^−1^ is the highest environmental concentration of AMPA that has been reported in soils^4^. A 1 mg.ml^−1^ aqueous AMPA stock solution was prepared using 10 mg of AMPA in 10 ml of water. This stock solution was used to obtain the nominal doses 100 (AMPA100), 250 (AMPA250), 500 (AMPA500), 750 (AMPA750), 1,000 (AMPA1000) and 2,500 (AMPA2500) μg.kg^−1^. The spiking of the pre-moistened TAS at the desired doses was performed by dilution of AMPA in the deionized water volume equivalent to 30% of the water holding capacity. In addition, a negative control with water, and a positive control with a 945.94 mg.kg^−1^ aqueous solution of boric acid^29^ were prepared and incubated together with the spiked treatments. The use of a reference substance as positive control is indicated in the ISO guidelines since it permits evaluation of the sensitivity of test organisms used over time. In standardized ecotoxicological tests with earthworms, the fungicide carbendazim is the recommended substance. However, boric acid is also a suitable reference substance for *E.fetida/E.andrei*^7^,^28^,^29^, so it was chosen for use in the present study. Boric acid causes a >50% reduction in abundance and biomass in field essays^28^ as well as a marked reduction in biomass gain and in juvenile production in laboratory essays, at doses similar to the one used in this study^29^.

### Acute toxicity test assay

The acute toxicity test was performed according to the ISO 11268-1 guideline^8^. Five plastic vessels with a fine mesh in their lids to allow gaseous exchanges were filled with 500 g of the corresponding contaminated TAS for each one of the eight treatments. Each vessel received 10 earthworms, previously weighed and washed with deionized water. Each treatment had five replicates, totalling 400 specimens overall (8 treatments × 5 replicates × 10 individuals). The vessels were closed and kept in the dark at 20 ± 2 °C. After 7 days, dead earthworms were removed and the living individuals counted and weighed. All the specimens were again placed in the same vessels and after another 7 days (14 days total), dead earthworms were removed and the survivors counted and weighed for wet biomass determination.

### Chronic toxicity test assay

The chronic toxicity test was performed according to the ISO 11268-2 guideline^8^. As in the previous assay, five vessels were filled with 500 g of the spiked TAS according to each of the eight treatments and 10 worms placed in each vessel. As a food source, 5 g of horse manure (oven dry at 60 °C) was placed on the surface of the substrate in each vessel. The vessels were closed and kept in the dark at 20 ± 2 °C. Throughout the test, water and food were checked weekly and added when necessary. After 28 days adult earthworms were carefully removed and the living ones counted and weighed. Soil was placed again in the same vessels and incubated for another 28 days. After 56 days, cocoons and juveniles were carefully hand-sorted, counted and weighed.

### Data analyses

The biomass loss in the chronic and acute assays was expressed as the percentage of loss relative to initial weight, according to the formula: 100 − (W_x_*100)/W_0_, where W_0_ is the mean weight of the earthworms of each replicate (vessel) at the beginning of the assay and W_x_ is the mean weight of the earthworms found in each replicate at x days after the beginning of the assay.

A general linear model was used to evaluate the effect of the treatments on the assessed parameters (biomass loss, cocoon number and juvenile number). Error variance structure was modelled using treatment as grouping criteria and Var (Ident) of R’s nlme library as variance function. When significant differences were found in the general model, *a posteriori* tests were performed by the DGC^42^ or LSD tests. All statistical analyses were performed using the InfoStat software^43^, as it is a friendly interpreter of R software.

## Additional Information

**How to cite this article**: Domínguez, A. *et al.* Toxicity of AMPA to the earthworm *Eisenia andrei* Bouché, 1972 in tropical artificial soil. *Sci. Rep.* **6**, 19731; doi: 10.1038/srep19731 (2016).
